# Supplementary material for: Programmable antisense oligomers for phage functional genomics
Source: Nature. 2025 Sep 10;646(8087):1195–203. doi: 10.1038/s41586-025-09499-6 (PMC12571901; doi:10.1038/s41586-025-09499-6)

nature portfolio

https://doi.org/10.1038/s41586-025-09499-6

Supplementary information

Programmable antisense oligomers for phage functional genomics

In the format provided by the authors and unedited

Supplementary Data Fig. 1

Fig. 1c

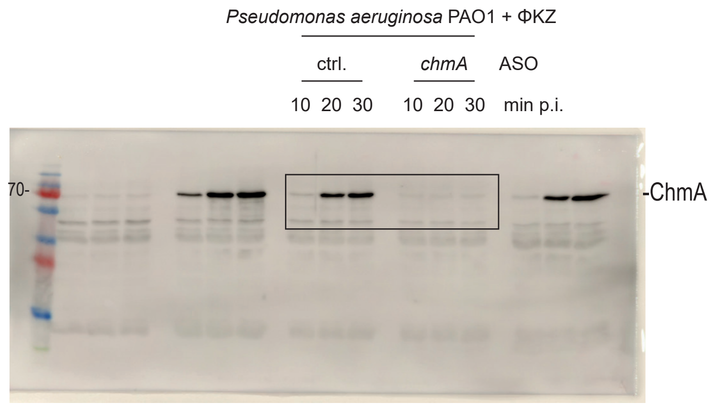

anti-ChmA

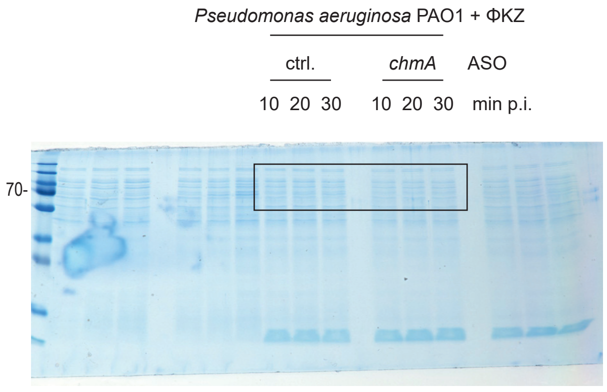

Coomassie

Fig. 4e

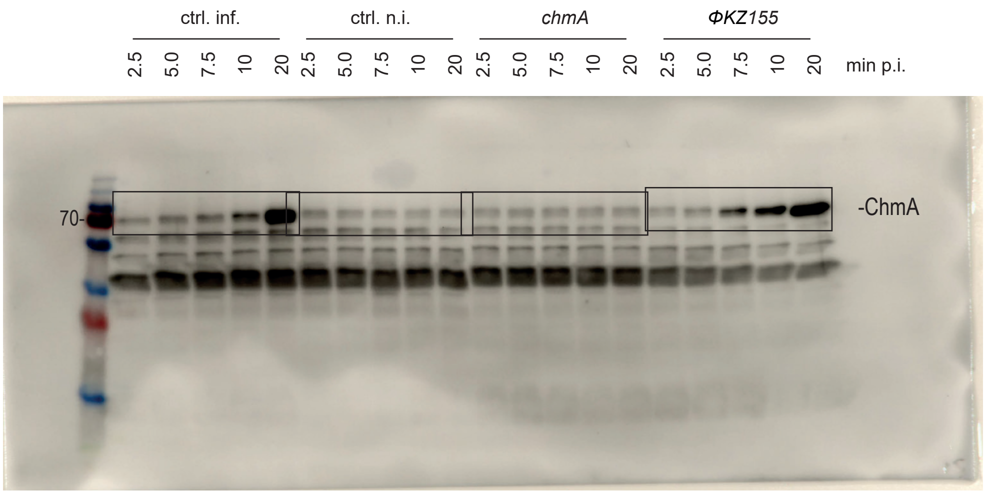

anti-ChmA

Fig. 4f

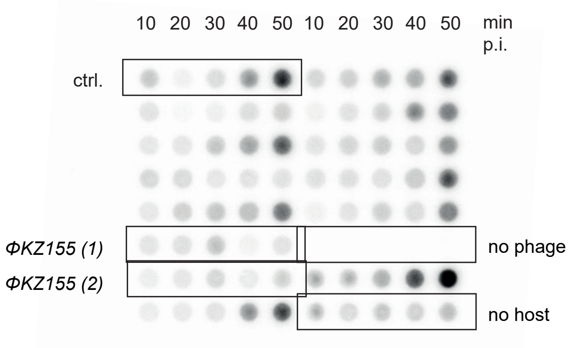

ΦKZ gDNA, ^{32}P-radiogram

Ext. Data Fig. 2c

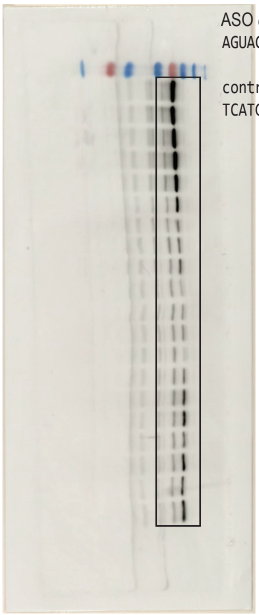

ASO *chmA* SD start codon
AGUACUUAGGUUCUUCUUGAUCAAGAAACCAUAAUCGUAAAGGUAUUUAAAAUGGCUGUUAACGAAAACGAAAUCGGCACCGUUCAAACUCAAGCUAC

control
TCATGAATCCA
AATCCAAGAAG
AAGAAGAACTA
GAACTAGTTCT
AGTTCTTTGGT
TCTTTGGTATT
TTGGTATTAGC
GTATTAGCATT
TTAGCATTTCC
TAGCATTTCCA
AGCATTTCCAT
> GCATTTCCATA < primary ASO in study
CATTTCCATAA
ATTTCCATAAA
TTTCCATAAAT
TTCCATAAATT
TCCATAAATTT
CCATAAATTTT
CATAAATTTTA

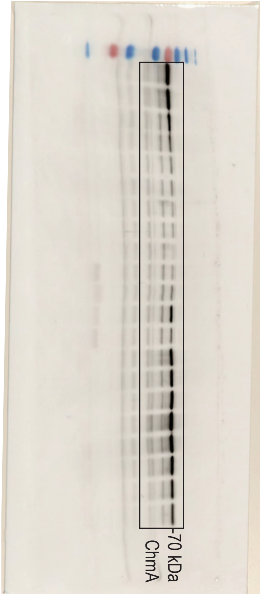

anti-ChmA

ATAAATTTTAC
TAAATTTTACC
AAATTTTACCG
AATTTTACCGA
ATTTTACCGAC
TTTTACCGACA
TTTACCGACAA
TTACCGACAAT
TACCGACAATT
ACCGACAATTG
CCGACAATTGC
CGACAATTGCT
GACAATTGCTT
AATTGCTTTTG
TGCTTTTGCTT
TTTTGCTTTAG
TGCTTTAGCCG
TAGCCGTGGCA
GTGGCAAGTTT
AAGTTTGAGTT
TGAGTTCGATG

Ext. Data Fig. 4b

	PaLo8		-9		-39		-44	
ASO	ctrl.	*chmA*	ctrl.	*chmA*	ctrl.	*chmA*	ctrl.	*chmA*

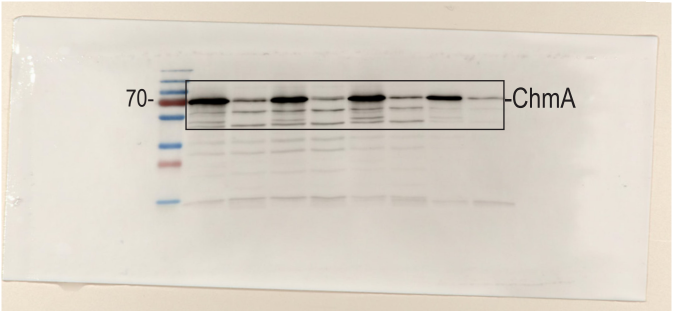

anti-ChmA

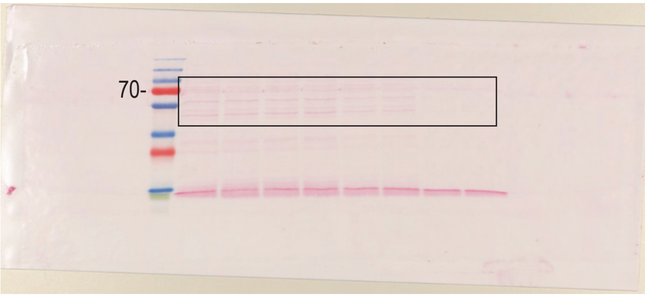

Ponceau S

Ext. Data Fig. 5b

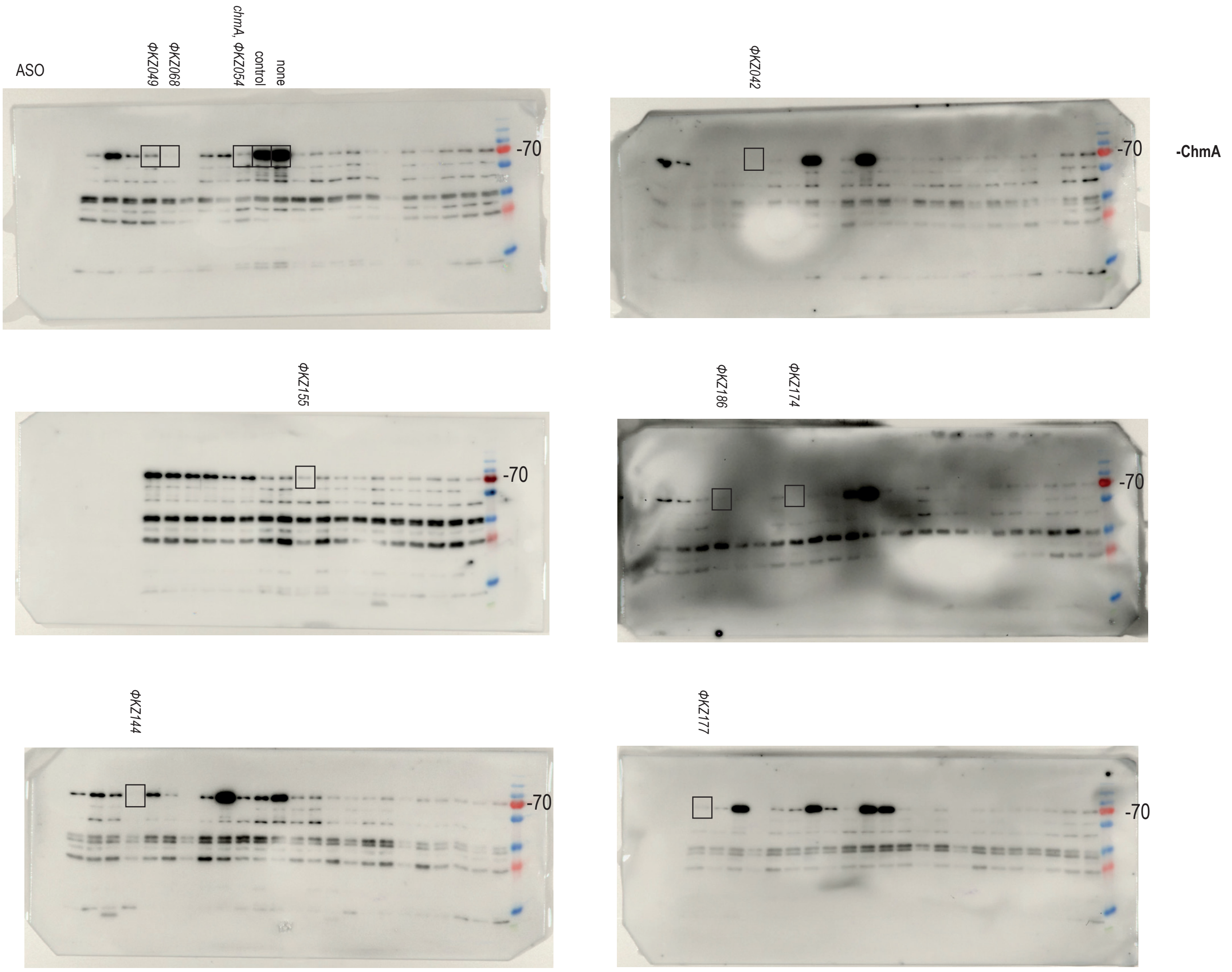

anti-ChmA

Ext. Data Fig. 5c

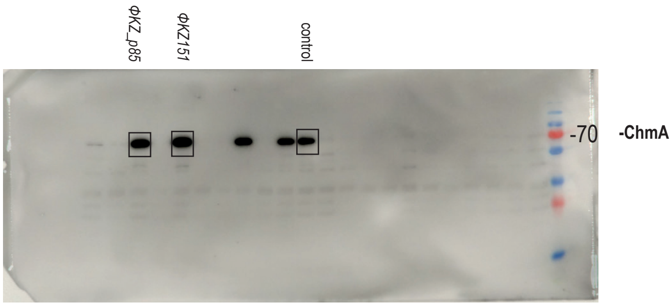

anti-ChmA

Ext. Data Fig. 8d

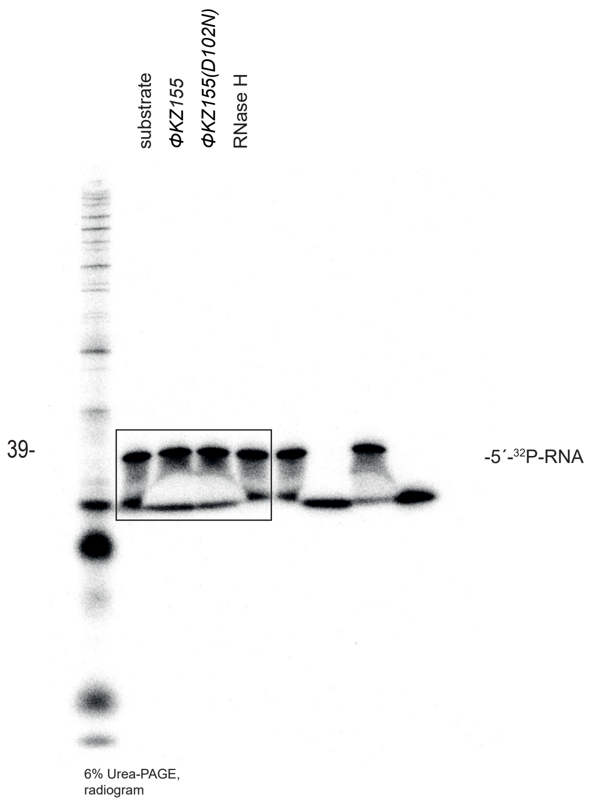

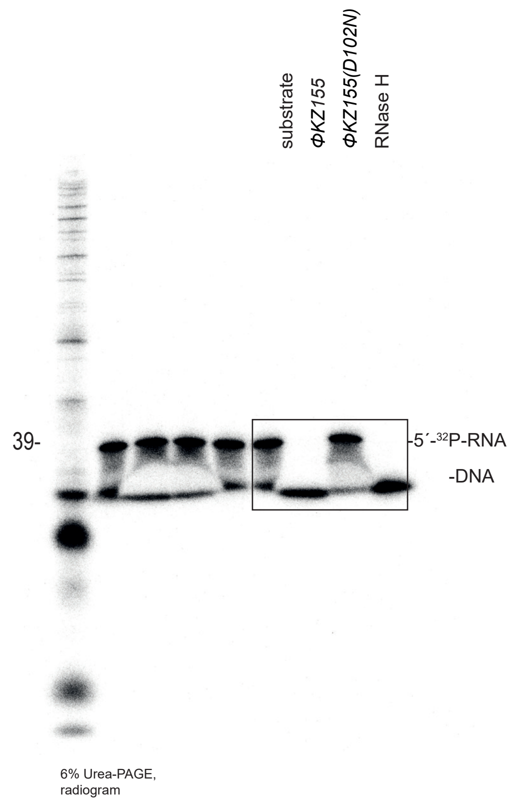

Ext. Data Fig. 8g

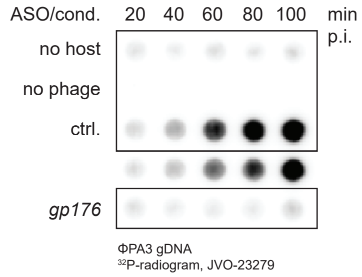

Ext. Data Fig. 8h

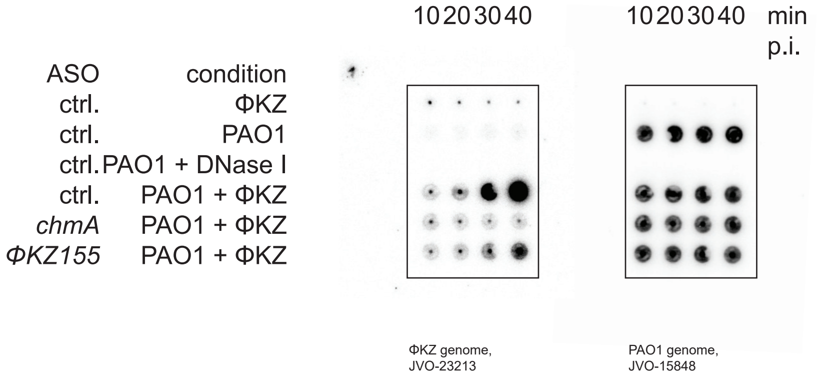

Supplement: Supplementary file 1 — Uncropped images of gels and blots. Ten display items for Figs. 1c and 4e,f and Extended Data Figs. 2c, 4b, 5b–d and 8g,h, showing uncropped and unprocessed blot, dot blot and gel source data with input references, protein or nucleotide size ladders, with relevant samples indicated. Black boxes around bands and dots indicate how the image was cropped for the final figure. [file 41586_2025_9499_MOESM1_ESM.pdf]
