# Supplementary material for: Programmable antisense oligomers for phage functional genomics
Source: Nature. 2025 Sep 10;646(8087):1195–203. doi: 10.1038/s41586-025-09499-6 (PMC12571901; doi:10.1038/s41586-025-09499-6)
Supplement: Supplementary file 2 — Reporting Summary [file 41586_2025_9499_MOESM2_ESM.pdf]

nature portfolio

Corresponding author(s): Jörg Vogel

Last updated by author(s): Jul 24, 2025

Reporting Summary

Nature Portfolio wishes to improve the reproducibility of the work that we publish. This form provides structure for consistency and transparency in reporting. For further information on Nature Portfolio policies, see our Editorial Policies and the Editorial Policy Checklist.

Statistics

For all statistical analyses, confirm that the following items are present in the figure legend, table legend, main text, or Methods section.

n/a	Confirmed	
☐	☒	The exact sample size (*n*) for each experimental group/condition, given as a discrete number and unit of measurement
☐	☒	A statement on whether measurements were taken from distinct samples or whether the same sample was measured repeatedly
☐	☒	The statistical test(s) used AND whether they are one- or two-sided<br>*Only common tests should be described solely by name; describe more complex techniques in the Methods section.*
☒	☐	A description of all covariates tested
☒	☐	A description of any assumptions or corrections, such as tests of normality and adjustment for multiple comparisons
☐	☒	A full description of the statistical parameters including central tendency (e.g. means) or other basic estimates (e.g. regression coefficient) AND variation (e.g. standard deviation) or associated estimates of uncertainty (e.g. confidence intervals)
☐	☒	For null hypothesis testing, the test statistic (e.g. *F*, *t*, *r*) with confidence intervals, effect sizes, degrees of freedom and *P* value noted<br>*Give P values as exact values whenever suitable.*
☒	☐	For Bayesian analysis, information on the choice of priors and Markov chain Monte Carlo settings
☒	☐	For hierarchical and complex designs, identification of the appropriate level for tests and full reporting of outcomes
☒	☐	Estimates of effect sizes (e.g. Cohen's *d*, Pearson's *r*), indicating how they were calculated

Our web collection on statistics for biologists contains articles on many of the points above.

Software and code

Policy information about availability of computer code

Data collection	no software was used for data collection
Data analysis	Image J 1.53, READemption 0.4.3 and 2.0.4, DeSeq2 1.44.0, Integrated Genomic Viewer (IGV) 2.18.4, MaxQuant Perseus 2.1.3.0, Alphafold 3 server (no version given, https://alphafoldserver.com)

For manuscripts utilizing custom algorithms or software that are central to the research but not yet described in published literature, software must be made available to editors and reviewers. We strongly encourage code deposition in a community repository (e.g. GitHub). See the Nature Portfolio guidelines for submitting code & software for further information.

Data

Policy information about availability of data

All manuscripts must include a data availability statement. This statement should provide the following information, where applicable:

- Accession codes, unique identifiers, or web links for publicly available datasets
- A description of any restrictions on data availability
- For clinical datasets or third party data, please ensure that the statement adheres to our policy

The genome annotation files used for ASO design are available at the National Center for Biotechnology Information (NCBI) (https://www.ncbi.nlm.nih.gov/) under accession numbers NC_004629.1 (ΦKZ), NC_028999.1 (ΦPA3), NC_001628.1 (PP7), NC_011421.1 (SPO1), NC_041973.1 (RAY). Raw sequencing data and coverage files have been deposited at Gene Expression Omnibus68 for ChmA knockdown and the screen with accession numbers GSE269401 and GSE269911, respectively.

Proteomics data have been deposited at PRIDE69 with the identifier PXD062538. Processed data are provided in Suppl. Data 1-4. Materials are listed in Suppl. Data 5. Source data for Fig. 3a, b, Ext. Data Fig. 2a, 3a, b, and 6a are provided with this paper. Uncropped images of blots are available in Supplementary Figure 1. Other source data are deposited at Zenodo (doi: 10.5281/zenodo.16357062).

Research involving human participants, their data, or biological material

Policy information about studies with human participants or human data. See also policy information about sex, gender (identity/presentation), and sexual orientation and race, ethnicity and racism.

Reporting on sex and gender	n/a
Reporting on race, ethnicity, or other socially relevant groupings	n/a
Population characteristics	n/a
Recruitment	n/a
Ethics oversight	n/a

Note that full information on the approval of the study protocol must also be provided in the manuscript.

Field-specific reporting

Please select the one below that is the best fit for your research. If you are not sure, read the appropriate sections before making your selection.

☒ Life sciences ☐ Behavioural & social sciences ☐ Ecological, evolutionary & environmental sciences

For a reference copy of the document with all sections, see nature.com/documents/nr-reporting-summary-flat.pdf

Life sciences study design

All studies must disclose on these points even when the disclosure is negative.

Sample size	No sample size calculation was performed. For bacterial assays a sample size of 2 was chosen per condition in independent experiments. Based on experience this is sufficient to make conclusions about effects in the range of more than 2-fold.
Data exclusions	We did not observe outliers in our data that needed to be excluded.
Replication	Bacterial assays were completed in minimum two independent experiments and the error accounts were reported. ASO experiments were conducted multiple times in independent experiments and/or the gene was targeted with multiple ASOs with a similar outcome. Top hits were reproduced twice at minimum. Biochemical experiments were reproduced twice at minimum in independent experiments with similar outcomes. The sequencing screen assay was conducted once/condition, because we were able to validate the results for the top hits that showed strong phenotypes through comparing two time points at 15 and 30 min per targeted gene. All attempts to replicate data were successful.
Randomization	This study did not require randomization. Covariates in microbiological experiments were controlled by restricted and well characterized genotypes (e.g. PAO1) of the strains, identical inoculum, media, growth conditions related to temperature, shaking, and incubation times. The phage stocks were produced in large amounts and tested for titer frequently to ensure reproducibility. ASOs were freshly diluted in a standardized procedure described in the methods section. With this approach, we were able to reduce the systematic influence of covariance.
Blinding	This study does not involve procedures that require blinding. The experimental procedures were standardized and the readout of the data was not based on personal assessment. All data were evaluated in raw format by multiple scientists. Microscopy, plaque and biochemical experiments were conducted and interpreted multiple times by different researchers based on raw data. Blinding was not considered critical due to the objective and quantitative nature of the experimental measurements (e.g., CFU/PFU counts, or enzymatic activity assays). In case of microscopy, we aimed to image in one view many cells with infection ratios >90% that resulted in similar phenotypes for cell morphology and inner structures and did not require user-based selection of individual cells. In addition, the selection of the zoom-in images was conducted by other scientists than the ones who recorded the images.

Reporting for specific materials, systems and methods

We require information from authors about some types of materials, experimental systems and methods used in many studies. Here, indicate whether each material, system or method listed is relevant to your study. If you are not sure if a list item applies to your research, read the appropriate section before selecting a response.

Materials & experimental systems

n/a	Involved in the study
☐	☒ Antibodies
☒	☐ Eukaryotic cell lines
☒	☐ Palaeontology and archaeology
☒	☐ Animals and other organisms
☒	☐ Clinical data
☒	☐ Dual use research of concern
☒	☐ Plants

Methods

n/a	Involved in the study
☒	☐ ChIP-seq
☒	☐ Flow cytometry
☒	☐ MRI-based neuroimaging

Antibodies

Antibodies used

Commercial antibody sera were generated by Eurogentec. Rabbits were immunised with the purified ChmA protein. The rabbit serum (no. 2481) was used in a 1:10,000 dilution together with anti-rabbit-HRP antibody (Thermo Scientific, 31460) in a 1:10,000 dilution in 5% BSA/TBST for ChmA detection in immunoblotting.

Validation

For anti-rabbit-HRP (Thermo Scientific, 31460), see the product information sheets of the manufacturer; this antibody also showed reliable and consistent results in previous publications from the Vogel lab with various ectopically expressed targets.

Anti-ChmA Antibody specificity was validated in immunoblotting by comparison between ΦKZ-infected and non-infected cells that yielded a defined band at 70 kDa corresponding for ChmA only in infected cells (Fig. 1c). In addition, we validated it after ASO-based ChmA knockdown, with this approach we were able to discriminate between specific and unspecific interactions.

Plants

Seed stocks

n/a

Novel plant genotypes

n/a

Authentication

n/a
