# Supplementary material for: Programmable antisense oligomers for phage functional genomics
Source: Nature. 2025 Sep 10;646(8087):1195–203. doi: 10.1038/s41586-025-09499-6 (PMC12571901; doi:10.1038/s41586-025-09499-6)
Supplement: Supplementary file 3 — Factors that affect ΦKZ plaque efficiency and ChmA levels. Target transcripts are listed with core genome blocks38, early infection transcript and protein abundance37. The PFU/CFU and ChmA level effect of each respective knockdown is depicted as no effect (), weak (+), effective (++), very effective plaque or ChmA level reduction (+++) and increased plaque or ChmA level (-), respectively. [file 41586_2025_9499_MOESM3_ESM.pdf]

Supplementary Table 1

CATEGORY	locus-tag ΦKZ-	core genome group	protein abundance	transcript abundance 10 min p.i.	3 rounds effect CFU/PFU	3 rounds effect IB anti-ChmA
**PHAGE NUCLEUS ASSOCIATED**						
ChmB nuclear shell protein	002				+	+
PhuZ tubulin protein	039				++	++
ChmA major nuclear shell protein	054	I			+++	+++
ChmC RNA-binding protein	067	II			++	+++
PicA nuclear shell import protein	069	II			+++	+++
**DNA/RNA METABOLISM**						
Dip protein - RNA degradosome inhibitor	037				+	++
DNA polymerase	050	I			++	++
Putative DNA-directed RNA polymerase beta subunit	055	I			+++	+++
HNH endonuclease	056				+	++
Putative nuclease	065	II			++	++
Putative nvRNAP (non-virion RNAP) sigma factor	068	II			++	+++
HNH endonuclease	072				+++	++
Putative RAD2/SF2 helicase	075	II				++
Putative DNA polymerase	082	other			+	+++
Putative DnaB helicase	118	V				
DNA endonuclease	137					
Putative ribonuclease HI	155	VI			+++	+++
Putative SbcC-like nuclease	165	V			+	+
Virion RNAP subunit	176	VI			+	+++
HNH endonuclease	179					
Ribonuclease H	199				+	+
DEAD/DEAH box helicase	203					+
Endonuclease	272					+
HNH endonuclease	296		n.d.			+
**CAPSID & TAIL**						
Internal head protein	094				+	+
Virion structural protein	119				++	++
Major capsid protein	120	V			+++	+++
Virion structural protein	126					+
Putative virion structural protein	129	V				+
Tail fiber protein	146				n.d.	n.d.
Tail sheath	145					
**OTHER ANNOTATED**						
Dihydrofolate reductase	004					
Ribosome-binding protein	014				+++*	+++*
Putative terminase large subunit function	025	VII				+
Macro domain-containing protein	104					+
Endolysine, transglycosylase	144				++	+++
Putative UvsX protein	152	VI			n.d.	n.d.
Putative lysozyme domain protein	181	VI				
Putative thymidylate kinase	188	V				
DprA-like DNA recombination-mediator protein	207					
AAA family ATPase	208					+
Thymidylate synthase	235				+	+
Ribonucleotide-diphosphate reductase subunit beta	305				++	+++
Ribonucleotide reductase	306				-	-
**UNCHARACTERISED CORE GENOME**						
	032	other				
	042	other			+++	+++
	049	I			++	+++
	052	I			+	++
	059	other			++	+++
	062	II				
	066	II			+	
	070	II				
	077	III			n.d.	n.d.
	079	III				+
	089	IV			++	++
	098	V			n.d.	n.d.
	100	V			n.d.	n.d.
	122	V			+	+
	140	V				
	147	VI				++
	153	VI				+
	161	VI				++
	171	VI			++	++
	174	VI			+++	+++
	177	VI			+++	+++
**EARLY EXPRESSED/CONSERVED/ABUNDANT**						
	064				+	+++
	124				+	++
	151				-	-
	154				++	++
	169				+	++
	186				+++	+++
	283					+
	286					++

core genome: I, II, III, IV, V, VI, VII, other

rel. prot. abundance (log_{10}iBAQ): 7 – 12

transcript abundance (TPM): 0 – 11E3

Supplementary Table 1 | Factors that affect ΦKZ plaque efficiency and ChmA levels. The association of target transcripts to Chimalliviridae core genome blocks[38], transcript abundance at 10 min p.i. and protein abundance (average values at 10, 20, 30 min p.i.) based on[37] are indicated. The PFU/CFU and ChmA level effect of each respective knockdown is depicted as no effect ( ), weak (+), effective (++), very effective plaque or ChmA level reduction (+++) and increased plaque or ChmA level (-), respectively; for details see **Extended Data Fig. 5a**, **Supplementary Data 2**. A minimum of two ASOs were tested per gene, the stronger effect is shown. Toxic ASOs were omitted. n.d. not determined, e.g. if both ASOs were toxic. *ΦKZ014 knockdown caused an abrogation of plaques only in the PaLo44 strain.
